# Analysis of Adaptive Olaparib Resistance Effects on Cisplatin Sensitivity in Triple Negative Breast Cancer Cells

**DOI:** 10.3389/fonc.2021.694793

**Published:** 2021-07-22

**Authors:** Ambikai Gajan, Ashapurna Sarma, Seongho Kim, Katherine Gurdziel, Gen Sheng Wu, Malathy P. Shekhar

**Affiliations:** ^1^ Karmanos Cancer Institute, Detroit, MI, United States; ^2^ Department of Oncology, Wayne State University School of Medicine, Detroit, MI, United States; ^3^ Genome Sciences Core, Wayne State University, Detroit, MI, United States; ^4^ Department of Pathology, Wayne State University School of Medicine, Detroit, MI, United States

**Keywords:** PARP, acquired resistance, BRCA1, RNA-seq, γH2AX foci, RAD51

## Abstract

Poly-(ADP)-ribose polymerase inhibitors (PARPi) and platinum-based drugs are promising therapies for triple negative breast cancers (TNBC) with *BRCA1* or *BRCA2* loss. PARPi(s) show better efficacies when combined with platinum-based therapy, however, acquisition of PARPi resistance has been linked with co-resistance to platinum-based drugs. Here, we show that TNBCs with constitutively hyperactivated PARP-1 display greater tolerances for the PARPi olaparib and cisplatin, and respond synergistically to olaparib/cisplatin combinations with increased cytotoxicity. Regardless of *BRCA1* and PARP-1 activity status, upon gaining olaparib resistance (OlaR), OlaR MDA-MB-468 (*BRCA1* wild-type) and SUM1315 (*BRCA1* mutant) TNBC cells retain cisplatin sensitivities of their isogenic parental counterparts. OlaR TNBC cells express decreased levels of PARP-1 and Pol η, a translesion-synthesis polymerase important in platinum-induced interstrand crosslink repair. Although native RAD51 recombinase levels are unaffected, anti-RAD51 immunoreactive low molecular weight sbands are exclusively detected in OlaR cells. Despite normal *BRCA1*, RAD51 foci formation/recruitment to double-strand breaks are impaired in OlaR MDA-MB-468 cells, suggesting homologous-recombination impairment. RNA-seq and pathway analysis of cisplatin-affected genes revealed enrichment of G2/M cell cycle regulation and DNA repair pathways in parental and OlaR MDA-MB-468 cells whereas parental and OlaR SUM1315 cells showed enrichment of inflammatory stress response pathways associated with TNFR1/2, TWEAK and IL-17 signaling. These data show that TNBC models with wild type versus mutant *BRCA1* exhibit differences in CDDP-induced cellular response pathways, however, the CDDP-induced signaling responses remain stable across the isogenic models of OlaR from the same lineage. These data also show that adaptive OlaR does not automatically promote cisplatin resistance, implicating the potential benefit of platinum-based therapy for OlaR TNBCs.

## Introduction

Management of triple negative breast cancers (TNBCs) is a challenge to clinicians because of the absence of targeted therapies, their aggressive behavior, and poor outcomes. PolyADP ribose polymerase (PARP) inhibitors (PARPi) and platinum-based drugs are promising therapies for TNBC as they exploit homologous recombination (HR) repair defects in *BRCA*-associated TNBCs for “synthetic tumor lethality” (1). The efficacy of PARPi is studied as a monotherapy, in combination with cytotoxic therapies including platinum, or as a maintenance therapy in metastatic TNBC patients with germline *BRCA* mutations. PARPi(s) have shown better efficacies when combined with cisplatin (CDDP) or carboplatin (2, 3).

PARP belongs to the family of ADP-ribosyltransferases and catalyzes the addition of single or polyADP ribose (PAR) moieties onto target proteins. Among the 18 PARP family members, PARP-1 is the principal responder to DNA damage as it rapidly reaches the target damage site and mounts a robust catalytic activation response that influences cellular outcomes to the damage (4, 5). PARP-1 then forms polymers of ADP-ribose (PAR) that PARylates itself (autoPARylation) and other proteins to control cell growth, transcription, apoptosis, and DNA repair (6–9). PARP-1 accounts for 75-95% of PARylation activity as compared to 5-15% by PARP-2 (10–12). Thus, the PARylation status of PARP-1 is a good indicator of PARP activity. Although the exact role of PARP-1 in base excision repair (BER) is unclear, its accepted role is in accumulation of PAR chains that help recruit proteins involved in repair of single strand DNA breaks (SSB) as disruption of this activity causes delayed repair and sensitivity to agents that induce DNA strand breaks (13). PARPi traps PARP at damaged DNA, and the trapped PARP-DNA complexes are considered to be more cytotoxic than unrepaired SSBs caused by PARP inactivation (14, 15).

The PARPi, olaparib inhibits both PARP catalytic and DNA trapping activities (16), and has been approved for treatment of patients with germline *BRCA* positive Her2 negative metastatic breast cancer (17). Clinical efficacy was seen in patients with platinum exposure; however, the trial was not designed to assess olaparib efficacy in platinum resistant disease (17). Since TNBCs are increasingly being treated with platinum compounds, it is likely to influence the way PARPi(s) are used. PARPi resistance can evolve by (a) increased homologous recombination (HR) capacity, (b) altered nonhomologous end joining (NHEJ) repair capacity, (c) decreased levels/activity of PARP-1, or (d) decreased intracellular availability of the PARPi (18). Increase in activity of RAD51, a key HR protein, has been implicated in PARPi resistance (19, 20). Mechanisms restoring HR such as reverse mutation or gene conversion that restores the open reading frame in *BRCA1/2* have been proposed as another mechanism of PARPi resistance (21). Similarly, restoration of *BRCA* functionality by secondary reverse mutations has been proposed to contribute to platinum therapy resistance, however, the degree of overlap between PARPi and platinum resistance is not known.

Driven by data that platinum-based drugs and PARPi(s) are part of the therapeutic armamentarium for *BRCA1/2* mutated TNBCs, we addressed the question whether PARPi and CDDP sensitivities of TNBCs are related to their intrinsic PARP-1 activity status and/or their *BRCA1* status. Using isogenic TNBC models, we also determined the impact of adaptive PARPi resistance on CDDP sensitivities and whether this is influenced by TNBC *BRCA1* status. Here, we show that TNBC cells that have high intrinsic PARP-1 activities, regardless of their *BRCA1* status, display higher tolerance thresholds for both olaparib and CDDP, and respond synergistically with increased cell death when treated with olaparib and CDDP combination. TNBC cells with low PARP-1 activity do not benefit from olaparib/CDDP combination therapy because of their greater sensitivities to olaparib and CDDP. We also show that regardless of the *BRCA1* status, TNBC subtype or their PARP-1 activity status, upon acquisition of olaparib resistance (OlaR), OlaR TNBC cells express catalytically inactive and decreased levels of PARP-1. OlaR TNBC cells retain CDDP sensitivities as their parental counterparts, suggesting uncoupling of PARP-1 and CDDP repair pathways in PARPi resistant cells. RNA-seq transcriptome and Ingenuity Pathway analysis (IPA) identified CDDP-induced upregulation of GADD45 signaling, G2/M DNA damage checkpoint regulation and *BRCA1* DNA damage response signaling in parental and OlaR *BRCA1* wild type MDA-MB-468 TNBC cells. Similar analysis revealed upregulation of TNFR1/2 signaling, TWEAK (TNF related weak inducer of apoptosis) and IL-17 signaling in parental and OlaR *BRCA1* mutant SUM1315 TNBC cells. These data suggest that although *BRCA1* wild type and mutant TNBC cells display distinct CDDP induced cellular responses, these response pathways are not impacted by adaptive OlaR, strengthening the relevance of platinum-based therapy for management of PARPi resistant TNBCs.

## Materials and Methods

### Cell Lines and Cell Culture

*BRCA1* wild type MDA-MB-468 and MDA-MB-231, and *BRCA1* mutant HCC1937 TNBC cells were purchased from ATCC (Manassas, VA). *BRCA1* mutant SUM1315 TNBC cells were purchased from Asterand (Detroit, MI). Isogenic cells resistant to olaparib, were generated by exposing MDA-MB-468 or SUM1315 cells to gradually increasing doses of olaparib starting from 1 to 25 µM over four to six months. To ensure sustained drug resistance, the generated OlaR cell cultures, MDA-MB-468 OlaR and SUM1315 OlaR, were continuously maintained in media supplemented with 15 µM or 25 µM olaparib, respectively. Since complete depletion of olaparib from these cells is not possible and in order to minimize variability, OlaR cells maintained under olaparib exposure were used in all experiments instead of rinsed cells as controls. All cell lines were maintained in Dulbecco’s Minimal Essential Medium/F12 (DMEM/F12; Invitrogen, Carlsbad, CA) supplemented with 5% fetal bovine serum and all cell cultures were used within 5-10 passages.

### Cell Survival and Colony Forming Analyses

MDA-MB-231, HCC1937, MDA-MB-468 or SUM1315 TNBC cells and the OlaR MDA-MB-468 or SUM1315 counterparts were seeded at 3.5 - 7 × 10^3^ cells/well in 96-well plates and treated individually with olaparib or CDDP at 0-25 μM concentrations or together at equimolar concentrations in 1:1 constant ratio. Cell viability was assessed at 72 h post treatment by MTT assays. Experiments were performed in triplicates and results presented are representative of three independent experiments. Olaparib and CDDP interaction was determined using isobologram and combination index (CI) values calculated using CompuSyn software (Combosyn Inc., Paramus, NJ, USA). CI < 1, CI = 1, and CI > 1 indicate synergistic, additive, and antagonistic effects, respectively (22, 23). For evaluation of colony forming potentials, parental and OlaR MDA-MB-468 and SUM1315 cells were treated overnight with vehicle, olaparib or CDDP at their respective IC25 or IC50 doses, or their combination, and reseeded in drug-free media at 100 - 500 cells per well in 24-well plates. Colonies were detected by crystal violet staining and assessed with GelCount™ Oxford Optronix and CHARM algorithm with a minimum diameter of 100 μm set as the threshold for colony classification. Colony forming efficiency was expressed relative to control cells.

### Western Blot Analysis

Whole cell lysates were prepared as previously described (24) from cells treated overnight with vehicle, olaparib (IC50 dose), CDDP (IC25 or IC50 doses), or their combination. Whole cell lysates were similarly prepared from OlaR MDA-MB-468 or SUM1315 cells maintained in 15 or 25 μM olaparib, respectively, with and without overnight treatment with CDDP. 50-100 µg of protein were subjected to 4-20% gradient SDS-PAGE and western blot analysis of PARP-1 (Cell Signaling, MA), PAR (Santa Cruz Biotechnology, TX), Pol η (Abcam, MA), RAD51 (Calbiochem, MA), and β-actin (Sigma-Aldrich Chemicals, MO). Protein levels relative to the loading control β-actin were quantified by Image J.

### Immunofluorescence Staining

Parental MDA-MB-468 and SUM1315 cells were seeded on coverslips and treated overnight with olaparib, CDDP, or their combination. The OlaR counterparts of MDA-MB-468 or SUM1315 cells maintained in 15 or 25 μM olaparib, respectively, were similarly seeded and treated with CDDP. Cells were fixed with 10% phosphate buffered formalin, permeabilized with methanol:acetone (1:1, v/v), and immunostained with PARP-1, γH2AX (BioLegend, CA) or RAD51 antibodies, and corresponding Texas Red or FITC-conjugated secondary antibodies (Molecular Probes, Eugene, OR). Nuclei were counterstained with 4’,6-diamidino-2-phenylindole (DAPI). Images were collected on an Olympus BX60 microscope equipped with a Sony high resolution/sensitivity CCD video camera and processed using CellSens software. The results represent data obtained from 50–75 cells in at least three-five fields.

### Whole Genome Expression Analysis by RNA-seq

To identify transcripts that are altered by OlaR acquisition and to assess the impact of OlaR on CDDP sensitivity, parental MDA-MB-468 and SUM1315 cells were treated overnight with 3 or 8 μM olaparib, respectively, CDDP (1 or 2.5 μM: MDA-MB-468, or 2 and 4 μM: SUM1315), their combination or untreated. OlaR MDA-MB-468 and SUM1315 cells maintained in 15 or 25 μM olaparib, respectively, were treated with CDDP at concentrations similar to their respective parental counterparts. Total RNA was isolated using the Trizol reagent kit (Invitrogen, Carlsbad, CA), and RNA-seq libraries were prepared using Lexogen’s QuantSeq 3’mRNA-seq Library Prep Kit (FWD for Illumina) from 200 ng of DNase I treated RNA. The barcoded libraries were multiplexed (in batches of 27) at equimolar concentrations and sequenced with 50 bp reads in rapid mode on an Illumina HiSeq 2500. RNA-seq analysis was conducted through the Genome Sciences Core at Wayne State University. Data were demultiplexed using Illumina’s CASAVA 1.8.2 software, and reads were aligned to the human reference genome (Build hg38) (25) and data analyzed using R/Bioconductor package edgeR (version 3.28.0) (26). For differential gene expression analysis, the edgeR function ‘glmQLFTest’ was used. FDR was computed using Benjamini-Hochberg method (27), and heatmap and hierarchical clustering were carried out using the R function heatmap.2 in the R package gplots (v3.0.1.1). Differentially expressed genes (DEGs) between olaparib treated parental and OlaR, olaparib and olaparib+CDDP, and between CDDP and olaparib+CDDP groups in parental and OlaR counterparts were detected using 5% FDR and fold-change (FC) of ≥2. Ingenuity Pathway Analysis (IPA, Ingenuity Systems, http://www.ingenuity.com) was performed to obtain biological information on the pathways perturbed by olaparib or CDDP treatment in parental *vs*. OlaR TNBC counterparts.

### Statistical Analysis

Comparisons between two individual groups or across three or more groups were done using two-tailed independent t-test, pairwise post-hoc analysis using Holm’s procedure, or one-way ANOVA. Experimental results are presented as the mean ± standard deviation (S.D.) or standard error of mean (S.E.M). Statistical significances were considered if P < 0.05. All statistical analyses were performed with GraphPad Prism and the statistical computing software R.

## Results

### Olaparib Sensitivity Is Not Correlated With *BRCA1* Status of TNBC Cells

**Figure 1:**
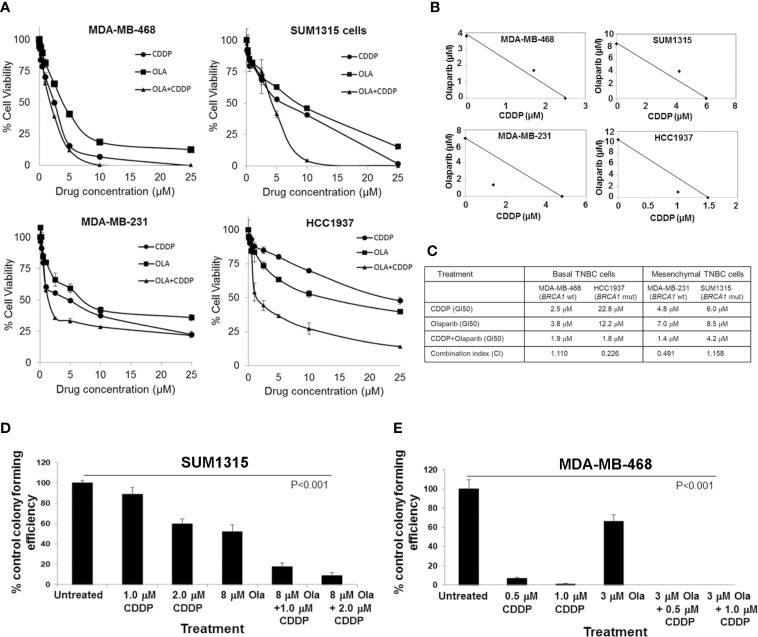
Olaparib and cisplatin (CDDP) *in vitro* sensitivities are not influenced by the BRCA1 status of TNBC cells. **(A)** MTT assays of MDA-MB-468, SUM1315, MDA-MB-231 and HCC1937 cells. Data are mean ± S.D. of three independent experiments. **(B)** Isobologram analysis of the combination of olaparib and CDDP in constant 1:1 ratio. The individual doses of olaparib and CDDP to achieve 50% growth inhibition (GI50) are plotted on the axes, and the position of the observed dose pair is indicated. Points falling below the line indicate synergism whereas those above the line indicate antagonism. **(C)** GI50 values for olaparib, CDDP, their combination (constant 1:1 ratio), and combination index (CI) values calculated using CompuSyn. **(D, E)** Clonogenic analysis of olaparib, CDDP and their combination at the indicated doses in SUM1315 **(D)** and MDA-MB-468 **(E)** cells. Results are expressed as mean ± S.D. (percent of control colony formation efficiency) from three independent experiments and analyzed by one-way ANOVA, P<0.001. Pairwise post-hoc analysis using Holm’s procedure was performed to compare effects caused by combination treatments to those caused by single treatment (**Supplementary Tables 1** and **2**).

We assessed olaparib and CDDP sensitivities of *BRCA1* wild-type MDA-MB-468, MDA-MB-231, and *BRCA1* mutant SUM1315 (185delAG) and HCC1937 (5382insC) TNBC lines by MTT assays. MDA-MB-231, SUM1315 and HCC1937 lines regardless of their *BRCA1* status tolerated relatively higher doses of both olaparib and CDDP, whereas MDA-MB-468 cells despite normal *BRCA1* expression showed decreased tolerance to both olaparib and CDDP (**Figure 1A**). The TNBC lines HCC1937, SUM1315 and MDA-MB-231 responded to olaparib/CDDP combination treatments with decreases in IC50 values whereas olaparib/CDDP combination produced no additional benefit in MDA-MB-468 cells likely due to their greater sensitivities to both olaparib and CDDP (**Figures 1A–C**). Combination index (CI) and isobologram analysis verified synergistic increases in drug sensitivities in MDA-MB-231 (CI = 0.491) and HCC1937 (CI = 0.226) cells, and lack of drug synergy in MDA-MB-468 (CI = 1.110) and SUM1315 (CI = 1.158) cells (**Figures 1B, C**). The results of MTT assays were further verified by colony forming assays. Colony forming potentials of SUM1315 and MDA-MB-468 cells were significantly inhibited by olaparib/CDDP combination treatment as compared to single treatments with olaparib or CDDP (**Figures 1D, E**, and **Supplementary Figure S2**, **Supplementary Tables 1** and **2**; P<0.001 by one-way ANOVA and pairwise post-hoc analysis using Holm’s procedure). These data show that olaparib and CDDP sensitivities of TNBC cells are not correlated with their *BRCA1* status, which is consistent with the findings of Cary et al. (28). The differences in drug responses are also not related to their *p53* status as all these TNBC lines express mutant *p53*.

### TNBC Cells With Intrinsic PARP-1 Hyperactivity Are Less Sensitive to Olaparib, and Benefit From Olaparib/CDDP Combination Treatment

**Figure 2:**
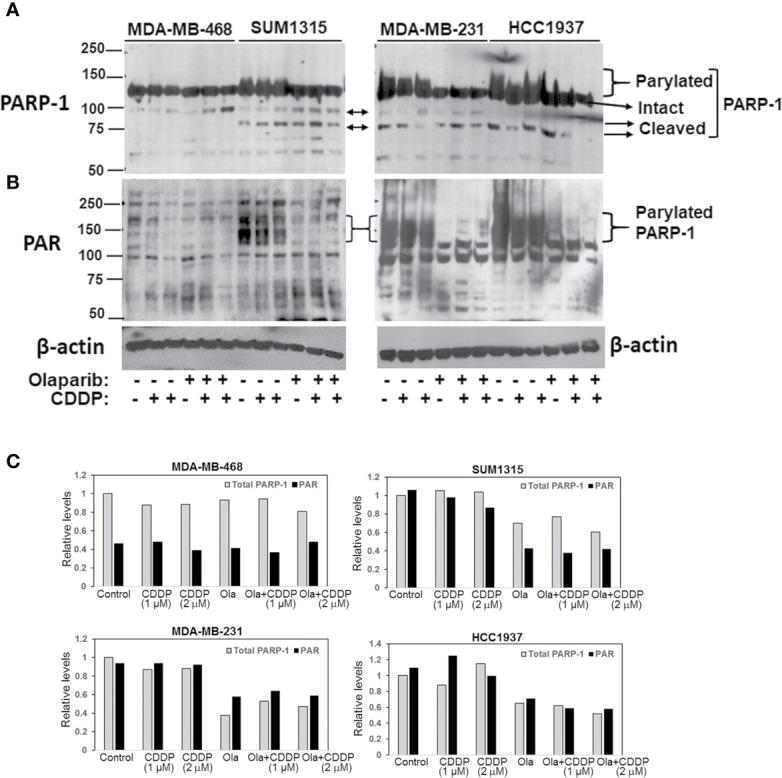
Analysis of TNBC intrinsic PARP-1 activities and regulation by olaparib and cisplatin (CDDP). Western blot analysis of PARP-1 **(A)** and PAR **(B)** in whole cell lysates of MDA-MB-468, SUM1315, MDA-MB-231 and HCC1937 cells treated overnight singly with olaparib or CDDP, or their combination. Cells were treated with 3 μM (MDA-MB-468), 8 μM (SUM1315, MDA-MB-231) or 10 μM (HCC1937) olaparib, 1 or 2 μM (MDA-MB-468), 2 or 4 μM (SUM1315, MDA-MB-231), or 4 and 8 μM (HCC1937) CDDP, or their combination. **(C)** Quantitation of relative total PARP-1 (native and PARylated) and PAR levels.

To determine if the differences in olaparib sensitivities between MDA-MB-231, SUM1315 and HCC1937 *vs*. MDA-MB-468 TNBC cells reflect inherent differences in PARP-1 activity, whole cell lysates prepared from control or cells treated singly with CDDP or olaparib, or their combination were analyzed by western blotting for PARP-1 and PARP-1 PARylation status using anti-PARP-1 and anti-PAR antibodies, respectively. Although the steady state levels of intact PARP-1 were similar in all lines, TNBC lines (SUM1315, MDA-MB-231 and HCC1937) that showed greater tolerance of olaparib displayed PARP-1 hyperactivation as evidenced by the presence of high molecular weight PARylated PARP-1 species in control cells (**Figure 2A**). PARP-1 activity was unaffected by CDDP as PARylated PARP-1 was detected with both PARP-1 (**Figure 2A**) and PAR (**Figure 2B**) antibodies. Olaparib treatment blunted PARP-1 activity as evidenced by loss of PARylated PARP-1 with both PARP-1 and PAR antibodies (**Figures 2A–C**). Although no further changes in PARP-1 modification were noted in cells treated with olaparib/CDDP combinations, the presence of 89 or 75 kDa cleaved PARP-1 was detected (**Figure 2A**). These data suggest that olaparib inhibition of PARP-1 activity renders it susceptible to proteolytic cleavage. Similar analysis of MDA-MB-468 cells showed negligible PARP-1 activity as evidenced by absence of PARylated PARP-1 using PARP-1 and PAR antibodies (**Figures 2A, B**). Treatment with olaparib/CDDP combination, however, increased PARP-1 cleavage as evidenced by increases in 89 kDa cleaved PARP-1 (**Figure 2A**). These differences in intrinsic PARP-1 activities between SUM1315, HCC1937 and MDA-MB-231 *vs*. MDA-MB-468 cells correlate with their olaparib and CDDP sensitivity profiles in **Figure 1**, further supporting the synergistic benefit of olaparib/CDDP combination treatment in TNBCs with PARP-1 hyperactivity.

### Generation of Isogenic Models of Olaparib Resistant TNBC Cells

**Figure 3:**
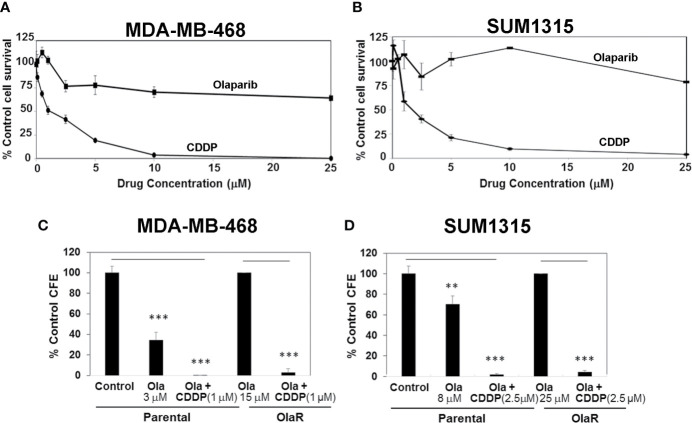
Olaparib resistant (OlaR) TNBC cells remain sensitive to cisplatin (CDDP). **(A, B)** Determination of olaparib and CDDP sensitivities of OlaR MDA-MB-468 **(A)** and SUM1315 **(B)** cells by MTT assays. **(C, D)** Clonogenic analysis of olaparib and CDDP sensitivities in parental and OlaR MDA-MB-468 **(C)** and SUM1315 **(D)** cells. Parental MDA-MB-468 and SUM1315 cells were treated with 3 or 8 μM olaparib, respectively. OlaR cells indicate MDA-MB-468 and SUM1315 cells maintained in 15 or 25 μM olaparib, respectively. Olaparib treated parental and OlaR MDA-MB-468 and SUM1315 cells were treated with 1 or 2.5 μM CDDP, respectively. Results are expressed as mean ± S.D. (percent of control colony formation efficiency) from three independent experiments. **P < 0.01, ***P < 0.001.

With the expanded use of PARPi(s) for TNBC management, development of acquired or adaptive resistance to PARPi is projected to limit their clinical efficacy. Even if an initial response is obtained, the majority of tumors subsequently become refractory to targeted agents after prolonged treatments due to development of adaptive resistance. We sought to develop OlaR TNBC cells since olaparib has shown promising anti-tumor activity in Her2/neu-negative metastatic breast cancer patients with *BRCA1* mutations (17) and is FDA approved for TNBC treatment. MDA-MB-468 and SUM1315 lines were chosen because they represent TNBC cells with wild type and mutant *BRCA1* status, respectively, and display notable differences in PARP-1 activity and olaparib sensitivities (**Figure 1**). To generate OlaR cells, the cells were gradually exposed to increasing concentrations of olaparib over several weeks. Cell survival was assessed every 3-5 weeks to verify increased tolerance thresholds for olaparib compared to their parental counterparts, and IC50 values were determined regularly. MTT assays confirmed the increased abilities of olaparib-adapted MDA-MB-468 and SUM1315 cells to tolerate olaparib as ~70% and >80% of MDA-MB-468 and SUM1315 cells, respectively, survived when exposed to 25 μM olaparib (**Figures 3A, B**) compared to their respective parental counterparts with IC50s of 3 and 8 μM, respectively (**Figure 1C** and **Supplementary Figure S1**). MDA-MB-468 and SUM1315 OlaR cells were thereafter continuously maintained in medium containing 15 or 25 µM olaparib, respectively.

### OlaR TNBC Cells Do Not Show Cross-Resistance to CDDP

Chemical and genetic interaction analysis have revealed strong correlations between PARPi and CDDP sensitivity and resistance profiles (29). Using the isogenic MDA-MB-468 and SUM1315 OlaR TNBC models, we tested whether OlaR acquisition contributes to simultaneous gain in CDDP resistance. The results from MTT assays show that both MDA-MB-468 and SUM1315 OlaR cells retain the CDDP sensitivities of their respective parental counterparts (**Figures 3A, B**). Colony forming assays were performed to verify these findings. MDA-MB-468 OlaR or SUM1315 OlaR cells continuously maintained in 15 μM or 25 µM olaparib, respectively, were exposed overnight to 1 µM (MDA-MB-468) or 2.5 µM (SUM1315) CDDP, rinsed and then seeded at 250 or 500 cells per well in drug-free medium. The colony forming potentials of both *BRCA1* wild type and *BRCA1* mutant OlaR derivatives were similarly inhibited by CDDP as their isogenic parental counterparts (**Figures 3C, D** and **Supplementary Figure S2**), suggesting uncoupling of OlaR and CDDP-induced responses.

### OlaR TNBC Cell Survival Involves PARP-1 and *BRCA1* Independent Pathways

**Figure 4:**
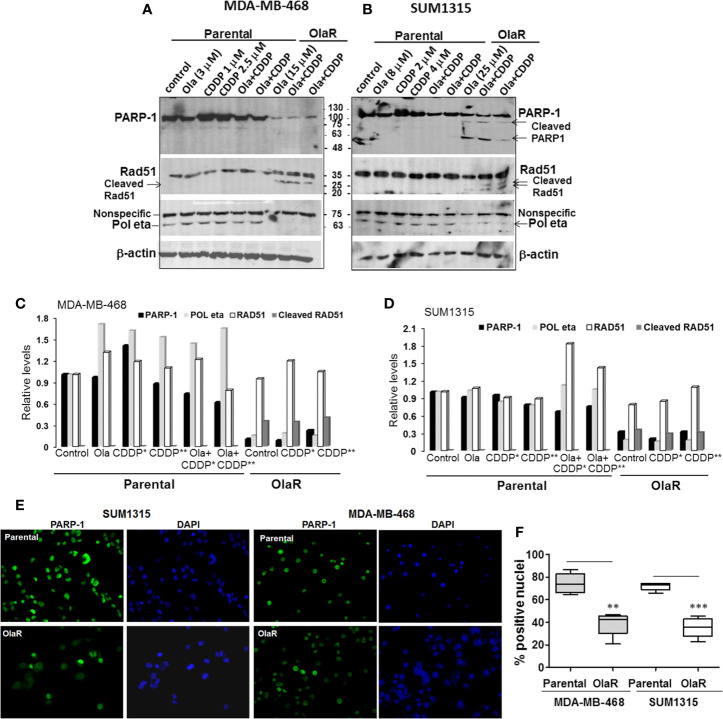
Olaparib resistant (OlaR) TNBC cells show downregulation of PARP-1 and POL η levels, and presence of cleaved RAD51. **(A, B)** Western blot analysis of the indicated proteins in whole cell lysates of MDA-MB-468 **(A)** and SUM1315 **(B)** cells treated overnight singly with olaparib or CDDP, or their combination. **(C, D)** Quantitation of the relative levels of PARP-1, Pol η and RAD51. Cleaved RAD51 was quantitated relative to native RAD51. **(E)** Immunofluorescence analysis of PARP-1 in parental and OlaR MDA-MB-468 and SUM1315 cells. Magnification ×400. **(F)** Quantification of cells with robust nuclear PARP-1 staining. At least 100 cells in four-five fields were scored, and results analyzed by 2-tailed Student’s t test. **P < 0.01, ***P < 0.001.

To gain insights into the molecular mechanisms of why a gain in adaptive OlaR did not confer CDDP resistance, we analyzed the effect of acquired OlaR on expression of its target PARP-1. Whole cell lysates were prepared from OlaR MDA-MB-468 and SUM1315 cells maintained in 15 or 25 μM olaparib, respectively, with or without CDDP (1 or 2.5 µM, MDA-MB-468, and 2 or 4 µM, SUM1315) treatment. Whole cell lysates were also prepared from parental MDA-MB-468 and SUM1315 cells treated singly with 3 or 8 µM olaparib, respectively, 1 or 2.5 µM CDDP (MDA-MB-468), 2 or 4 µM CDDP (SUM1315), or a combination of olaparib and CDDP. Western blot analysis of PARP-1 showed that the steady-state levels of PARP-1 were reduced ~3-5-fold, respectively, in OlaR SUM1315 and MDA-MB-468 cells as compared to their isogenic parental counterparts (**Figures 4A–D**). About 75 and 55 kDa cleaved PARP-1 fragments were observed in OlaR SUM1315 cells (**Figure 4B**), and CDDP treatment did not affect PARP-1 levels and processing (**Figures 4A, B**). Consistent with olaparib inhibition of PARP activity, PARP-1 expressed in OlaR cells is catalytically inactive as evidenced by loss of reactivity to PAR antibody (**Supplementary Figure S3**). Immunofluorescence staining showed robust PARP-1 nuclear expression in >70% of parental SUM1315 and MDA-MB-468 cells compared to 35-38% in their respective OlaR counterparts (**Figures 4E, F**). Since homologous recombination (HR) is implicated in both platinum and PARPi responses, we analyzed RAD51 levels as this recombinase is essential for HR repair. RAD51 steady-state levels were similar in both parental and OlaR counterparts, however, low molecular weight bands potentially reflecting cleaved RAD51 forms were exclusively detected in both OlaR models (**Figures 4A–D**). Similar analysis of Pol η, a translesion synthesis (TLS) DNA polymerase that interacts with RAD51 to stimulate D-loop extension for HR repair (30), showed ~5-6-fold decrease in both *BRCA1* wild type and mutant OlaR TNBC cells compared to their parental counterparts (**Figures 4A–D**). These data suggest that regardless of their *BRCA1* status or HR competence, both *BRCA1* wild type and mutant TNBC cells adapt to olaparib with downregulation of PARP-1 and Pol η, and potential RAD51 cleavage.

**Figure 5:**
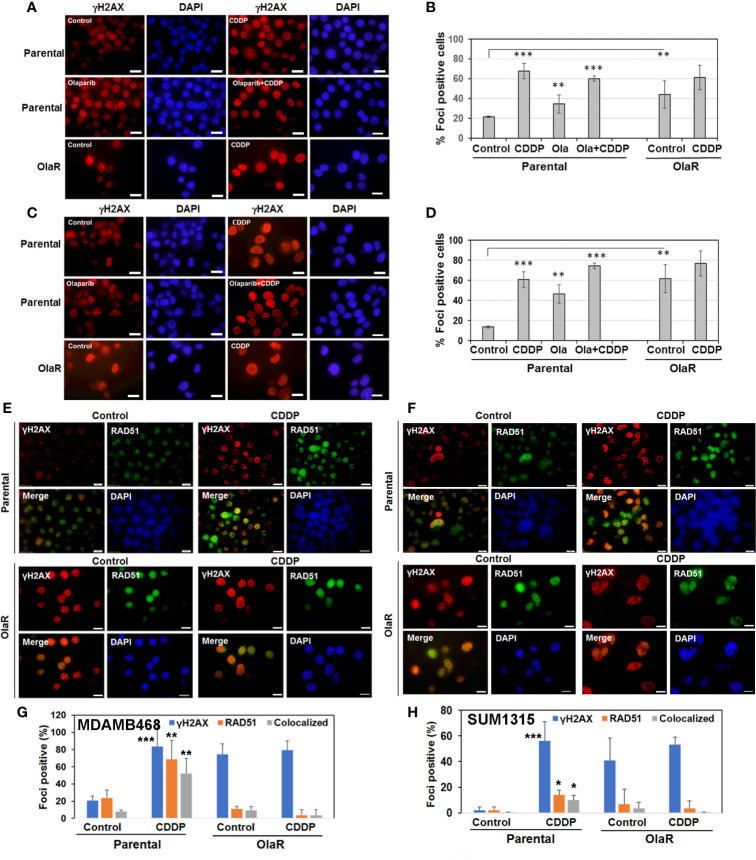
Olaparib resistance impedes Cisplatin (CDDP)-mediated induction of RAD51 foci formation and its colocalization with γH2AX foci in *BRCA1* wild type TNBC cells. Immunofluorescence staining of γH2AX **(A–D)** or γH2AX/RAD51 **(E–H)** foci in parental and OlaR MDA-MB-468 **(A, E)** or SUM1315 **(C, F)** cells treated with olaparib, CDDP or olaparib/CDDP combination. Scale bars, 20 μm. **(B, D, G, H)** Graphs show γH2AX or RAD51 foci positive cells scored from at least 100 cells in five-seven fields from two independent experiments by Image J. Results were analyzed by 2-tailed Student’s t test and only significantly affected groups indicated. *P < 0.05, **P < 0.01, ***P < 0.001.

Inductions of γH2AX and RAD51 foci formation are considered as response markers of double strand breaks (DSB) and HR repair, respectively. To compare parental and OlaR TNBC cell responses to CDDP, we performed immunofluorescence staining of γH2AX and RAD51. As indicated in **Figures 5A–D**, both OlaR MDA-MB-468 and SUM1315 cells, and their respective parental counterparts exposed to olaparib showed increases in γH2AX foci formation as compared to untreated parental controls, suggesting olaparib-induced DNA damage. Whereas CDDP treatment robustly increased γH2AX foci formation in both parental lines, it did not elicit similar responses in the OlaR cells potentially due to abundant DNA damage induced by uninterrupted exposure to olaparib (**Figures 5B, D**). Combination treatment with olaparib/CDDP did not further increase γH2AX foci formation in both parental and OlaR counterparts, suggesting saturation of DNA damage (**Figures 5B, D**). RAD51 foci formation was robustly induced by CDDP in parental MDA-MB-468 cells with ~50% colocalizing with γH2AX (**Figures 5E, G**). Despite robust γH2AX foci induction in OlaR MDA-MB-468 cells, RAD51 foci formation was significantly impaired and was not restored by CDDP treatment (P<0.001; **Figures 5E, G**). RAD51 was rather concentrated in nuclear bodies (visualized as pan-nuclear staining) without identifiable foci. Consistent with *BRCA1* requirement, RAD51 foci formation was only very weakly induced by CDDP in parental SUM1315 cells (**Figures 5F, H**), and as in OlaR MDA-MB-468 cells, OlaR SUM1315 cells showed pan-nuclear RAD51 staining without discrete foci (**Figures 5F, H**). These data suggest that continuous exposure to olaparib impairs HR function in OlaR MDA-MB-468 cells despite its normal BRCA1 status and that HR pathway may not be a major contributor of adaptive OlaR.

### Adaptive Olaparib Resistance Does Not Impact CDDP Response Pathways

**Figure 6:**
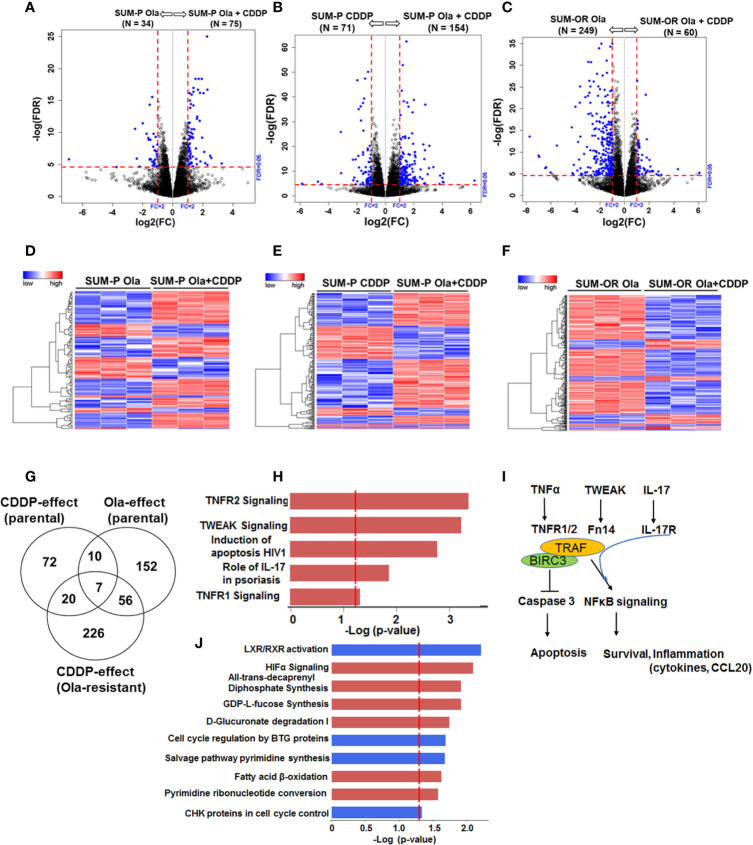
Olaparib resistant (OlaR) and parental *BRCA1* mutant SUM1315 TNBC cells respond to cisplatin (CDDP) with upregulation of inflammatory response pathways. RNA-seq analysis was performed on isogenic parental and OlaR SUM1315 TNBC cells. Parental cells were treated with 4 μM cisplatin (CDDP), 8 μM olaparib, or their combination. Note that the OlaR cells represent cells that were maintained in 25 μM olaparib without interruption and were used as such or treated with 4 μM CDDP. **(A–C)** Volcano plots. Each point in the volcano plot represents one gene. The blue and black dots represent differentially expression genes (DEGs) and unchanged genes, respectively. The horizontal and vertical dotted red lines indicate 5% FDR and fold-change (FC) of ≥2, respectively. The numbers (N) in parentheses at the top are the total number of DEGs that have larger expression levels in the corresponding group. **(D–F)** Hierarchical cluster maps; **(G)** Venn diagram of DEGs, 5% FDR and FC ≥2) and overlap in olaparib- and CDDP-induced genes in parental and OlaR counterparts. **(H)** CDDP impacted canonical pathways (red, upregulation) identified by IPA of 20 DEGs in the Venn diagram and **(I)** schematic representation of the affected pathways. **(J)** Canonical pathways affected by olaparib (red, upregulation and blue, downregulation) in parental cells identified by IPA corresponding to 152 DEGs in the Venn diagram.

**Figure 7:**
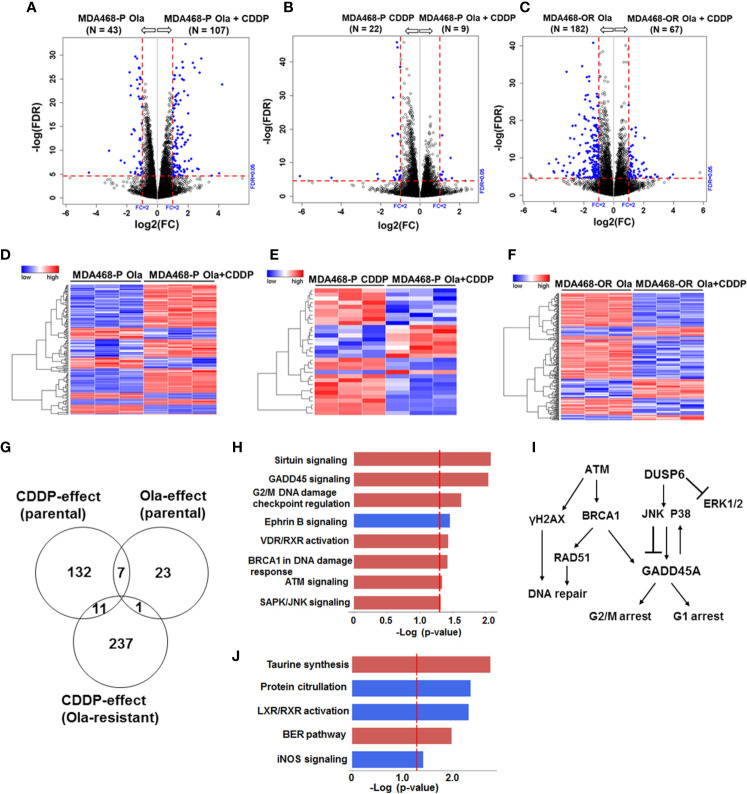
Olaparib resistant (OlaR) and parental BRCA1 wild type MDA-MB-468 TNBC cells respond similarly to cisplatin (CDDP) with upregulation of DNA damage response pathways. RNA-seq analysis was performed on isogenic parental and OlaR MDA-MB-468 TNBC cells. Parental cells were treated with 3 μM olaparib, 2.5 μM CDDP or their combination. The corresponding OlaR MDA-MB-468 cells represent cells maintained in 15 μM olaparib without interruption and were used as such or treated with 2.5 μM CDDP. **(A–C)** Volcano plots. Each point in the volcano plot represents one gene. The blue and black dots represent differentially expression genes (DEGs) and unchanged genes, respectively. The horizontal and vertical dotted red lines indicate 5% FDR and fold-change (FC) of ≥2, respectively. The numbers (N) in parentheses at the top are the total number of DEGs that have larger expression levels in the corresponding group. **(D–F)** Hierarchical cluster maps; **(G)** Venn diagram of DEGs, 5% FDR and FC ≥2) and overlap in olaparib- and CDDP-induced genes in parental and OlaR counterparts. **(H)** CDDP impacted canonical pathways (red, upregulation and blue, downregulation) identified by IPA of 11 DEGs in the Venn diagram and **(I)** schematic representation of the affected pathways. **(J)** Olaparib impacted pathways identified by IPA corresponding to 23 DEGs in the Venn diagram.

Our data thus far showed that regardless of their *BRCA1* status, the OlaR TNBC cells maintain their original (parental) CDDP sensitivities. Our data also suggest that upon acquisition of OlaR, repair of CDDP-induced DNA damage potentially involves BRCA1/RAD51 and PARP-1-independent pathways. To analyze the molecular underpinnings of PARP inhibition and adaptive OlaR on CDDP response, we characterized the global gene expression profiles by whole transcriptome sequencing and statistically significant differentially expressed genes (DEGs) identified in olaparib or CDDP treated parental and OlaR SUM1315 and MDA-MB-468 cells were subjected to Ingenuity pathway analysis (IPA) to identify the affected processes. Parental MDA-MB-468 or SUM1315 cells were untreated, or treated for 12 h singly with either olaparib (3 µM, MDA-MB-468 or 8 µM, SUM1315) or CDDP (1 µM or 2.5 µM for MDA-MB-468, or 2 µM or 4 µM for SUM1315), or their combination. OlaR MDA-MB-468 and SUM1315 counterparts continuously maintained in 15 or 25 µM olaparib, respectively, were treated with CDDP at concentrations similar to their parental counterparts. No gross morphological signs of cell death were detected under these treatment conditions, and cells were processed for RNA isolation and subsequent whole transcriptome sequencing. Volcano plot (**Figures 6A–C** and **Figures 7A–C**) and hierarchical cluster (**Figures 6D–F** and **Figures 7D–F**) analysis showed clear separations of DEGs between olaparib *vs*. olaparib/CDDP and CDDP *vs*. olaparib/CDDP treated parental and OlaR SUM1315 (**Figure 6**) and MDA-MB-468 (**Figure 7**) groups. Since treatment with the IC25 CDDP dose produced minimal effects on the transcriptome, they were not included in our analysis. Gene expression analysis identified 543 statistically significant DEGs (FDR ≤ 0.05) when comparing the olaparib and CDDP effects on parental and OlaR SUM1315 cells. Meta-analysis using Venn diagram revealed among the 543 DEGs (FDR ≤ 0.05 and absolute log2 FC ≥ 2), 93 genes overlapped between the olaparib and CDDP groups with 20 genes commonly expressed in CDDP treated parental and OlaR SUM1315 cells (**Figure 6G**). Pathway analysis of the 20 CDDP affected DEGs revealed enrichment of genes associated with TNFR1 and TNFR2 signaling, TWEAK (TNF related weak inducer of apoptosis) signaling, and interleukin-17 (IL-17) signaling networks in parental and OlaR SUM1315 cells, and involved BIRC3 (a member of the inhibitor of apoptosis proteins, IAPs), tumor necrosis factor receptor (TNFR)-associated factor TRAF1, and chemokine CCL20 among the top ranked genes (**Figures 6H, I** and **Supplementary Table S3**). Pathway analysis of the olaparib regulated 152 DEGs in parental SUM1315 cells (**Figure 6G**) identified downregulations in LXR/RXR signaling, cell cycle control by B-cell translocation gene (BTG) and CHK proteins, and salvage pathway of pyrimidine synthesis, and upregulation in pathways associated with HIF-1α signaling, GDP-fucose synthesis, D-glucuronate degradation and fatty acid β-oxidation (**Figure 6J** and **Supplementary Table S4**).

Similar analysis of olaparib and CDDP effects on parental and OlaR MDA-MB-468 cells revealed 411 statistically significant DEGs, and meta-analysis revealed among the 411 DEGs (FDR ≤ 0.05 and absolute log2 FC ≥ 2), 19 genes overlapped between the olaparib and CDDP groups and 11 genes were commonly expressed in CDDP treated parental and OlaR MDA-MB-468 cells (**Figure 7G**). IPA analysis of the 11 DEGs identified upregulation of sirtuin signaling, GADD45 signaling, G2/M DNA damage checkpoint regulation, *BRCA1* DNA damage response signaling, ATM, and SAPK/JNK signaling in parental and OlaR MDA-MB-468 cells, and involve GADD45A and DUSP6 as the predominant focus molecules (**Figures 7H, I** and **Supplementary Table S5**). Pathway analysis of olaparib affected 23 DEGs in parental MDA-MB-468 cells (**Figure 7G**) identified base excision repair (BER), a pathway known to be regulated by PARP, as well as pathways associated with LXR/RXR activation, protein citrullation, iNOS signaling and taurine synthesis (**Figure 7J** and **Supplementary Table S6**). Olaparib impacted the BER pathway only in MDA-MB-468 cells, suggesting a role for normal *BRCA1* function in DNA damage response after PARP inhibition. Our data also reveal that *BRCA1* wild type and mutant TNBC cells utilize fundamentally different signaling mechanisms to respond to CDDP induced damage and that TNBC cells with normal *BRCA1* function show enrichment of DNA damage response pathways. Taken together, these data show that a set of pathways impacted by CDDP are maintained in the corresponding parental and OlaR TNBC cells, and this is unaffected by their *BRCA1* status.

## Discussion

PARP inhibitors and CDDP exploit *BRCA* deficiencies to induce synthetic lethality. Thus, while PARP inhibition can enhance the effectiveness of platinum-based therapy, gain in PARPi resistance has been linked with co-resistance to platinum-based drugs. In this paper, we show that TNBC cells with constitutively high PARP-1 activities, regardless of their *BRCA1* status, display greater tolerances for both olaparib and CDDP, and respond synergistically to olaparib/CDDP combination therapy with increased cell death. In contrast, TNBC cells lacking PARP-1 activity display greater sensitivities to olaparib and CDDP, and show no additional benefit with combination therapy. The data also show that regardless of their initial PARP-1 activity status, upon acquisition of adaptive OlaR, TNBC cells express catalytically inactive and low levels of PARP-1. We also report the unexpected finding that both *BRCA1* wild type and mutant OlaR TNBC cells retain the CDDP sensitivities of their respective parental cells, suggesting uncoupling of PARP-1 and CDDP-induced damage repair pathways in OlaR cells.

PARP-1 constitutes >80% of overall PARP activity, and PARP-1 has been identified as a platinum–DNA damage response protein with affinity for both CDDP-induced intrastrand and interstrand DNA crosslinks (ICL) (29). Since proteins that recognize platinum-induced ICLs have also been identified in CDDP resistant cells (31), an important and continued role for PARP-1 in CDDP-induced DNA damage repair has been implicated. This is consistent with studies that showed PARPi(s) in combination with platinum-based drugs provide benefit not only to patients with germline *BRCA1/2* mutations but also for breast cancers having molecular characteristics of *BRCA1/2* mutant tumors or *BRCA*ness. The direct link between PARP-1 hyperactivity and CDDP resistance we observed in TNBC cells is consistent with similar findings from non-small cell lung cancer (NSCLC) cells (32) and strengthen their roles in BER and HR pathways (33). However, the retention of similar CDDP sensitivities in isogenic parental and OlaR TNBC cells observed in our study suggests that OlaR acquisition does not automatically confer CDDP resistance. Our data rather suggest that OlaR acquisition sets up a scenario wherein the PARP and CDDP regulated pathways can become disengaged.

Restoration of HR by secondary *BRCA1/2* reverse mutations or gene conversion and increased RAD51 activity are recognized mechanisms of PARPi resistance (21). However, our results contrast these findings as *BRCA1* wild type and mutant OlaR TNBC cells were rendered or remained HR-incompetent, respectively. Heatmap visualization of CDDP effects on RAD51 family members showed induction of RAD51 and RAD51AP1 transcripts in both TNBC models but not of the other RAD51 members (**Supplementary Figure S4**). However, notwithstanding the strong expression of native RAD51 protein (**Figure 4**) in OlaR cells, RAD51 foci formation is impaired in OlaR MDA-MB-468 cells despite their normal *BRCA1* status. Since RAD51 foci formation is robustly induced by CDDP treatment in MDA-MB-468 cells suggests that this loss of HR proficiency is specifically induced by olaparib-induced stress. In this regard, it is interesting to note that western blot analysis revealed the unique presence of low molecular weight potentially cleaved RAD51 forms only in OlaR MDA-MB-468 and SUM1315 cells but not in their isogenic parental counterparts. RAD51 cleavage by caspases in response to DNA damaging agents including ionizing radiation has been shown to decrease RAD51 strand exchange activity (34, 35). Proteolytic susceptibility of RAD51 is also affected by adenosine nucleotide binding as limited protease digestion generated low molecular weight (~15-20 kDa) forms of RAD51 only in the absence of ADP or ATP, suggesting an important role for ADP/ATP binding in RAD51 stabilization (36). While the mechanism of RAD51 cleavage in OlaR cells remains to be determined, it is plausible that these low molecular weight forms impact HR activity.

Western blot analysis also revealed decreases in PARP-1 and Pol η, a translesion synthesis (TLS) polymerase implicated in replication bypass across ICLs (37–40) and in D-loop DNA synthesis during HR *via* RAD51 interaction (30). Levels of γ-H2AX foci, a marker for DNA DSBs (41), are elevated in OlaR TNBC cells indicating constitutive olaparib-induced DNA damage. This suggests that OlaR TNBC cells are indeed capable of mounting robust DNA damage responses and that the HR pathway dysfunction is not due to defects in ATM-induced H2AX phosphorylation (41). PARP is activated at stalled replication forks and plays an important role in restarting stalled replication forks (42, 43). We postulate that the high levels of DNA damage sustained in OlaR cells due to PARP inhibition may overwhelm the DNA repair capacity that is required for restarting stalled forks. Since gain of OlaR does not result in simultaneous gain of CDDP resistance even in *BRCA1* wild type TNBC cells, our data suggest that *BRCA1* expression is not sufficient to ensure HR function necessary for efficient repair of CDDP induced ICLs under conditions of PARP inhibition in OlaR cells. The alterations such as Rad51 cleavage, and downregulations of Pol η and PARP-1 observed in OlaR TNBC cells could potentially result from sustained exposure to high doses of olaparib. However, since both MDA-MB-468 and SUM1315 OlaR TNBC models despite their difference in *BRCA1* status and exposure to variant levels of olaparib (the former to 15 μM olaparib *vs*. the latter to 25 μM olaparib), display similar olaparib-induced molecular changes suggest that the observed events are more likely due to acquired OlaR rather than random off-target effects. To exclude olaparib off-target effects, our efforts at establishing cells resistant to another PARPi, viz., talazoparib, were not successful because of its high inhibitory potency against TNBC cells. Since the increase in PARPi use will be paralleled by increases in the number of patients with acquired resistance to PARPi, our data shed light on potential effects of continued PARPi therapy regardless of whether they are direct or off-target. The exact mechanism for why PARP-1 levels are decreased in OlaR cells is not clear. Further studies are needed to determine whether the olaparib-induced sustained loss of PARP-1 catalytic activity and its autoPARylation renders the PARP-1 susceptible to proteasomal degradation.

Transcriptome analysis showed that *BRCA1* wild type and mutant TNBC cells coopt disparate signaling pathways to process CDDP-induced DNA damage. Pathway analysis of the CDDP-affected DEGs in parental and OlaR MDA-MB-468 cells revealed an overlapping role for GADD45A in regulating pathways associated with sirtuin signaling, cell cycle regulation, DNA repair and JNK signaling (**Figure 7I**). GADD45 proteins achieve cell cycle arrest by physically interacting with several proteins that regulate G1 and G2/M cell cycle arrest. They not only modulate the activity of stress kinases but their expression is also under the control of p38 and JNK MAPKs (44, 45), creating a feedback regulatory loop. Sirtuins are NAD^+^-dependent enzymes that deacetylate histones and non-histone proteins, and members of sirtuin family SIRT1, SIRT6 and SIRT7 modulate DNA repair pathways at multiple pathways including BER, NER, HR and NHEJ besides regulating cell survival, senescence, death, and differentiation (46). Although we observed no significant alterations in expression of sirtuins, upregulation of sirtuin signaling is associated with GADD45A and DUSP6 (a strong negative regulator of ERK1/2 signaling (47), as the regulatory molecules. Similar analysis of CDDP affected DEGs in parental and OlaR SUM1315 cells revealed enrichment of pathways associated with TNFR1/2, TWEAK and IL-17 signaling and involved BIRC3, TRAF1 and CCL20 genes (**Figure 6I**). BIRC3 and TNFR-associated factor TRAF1 play pivotal roles in regulation of nuclear factor-κB (NF-κB) signaling and apoptosis. Complex formation between TNFR-associated factors TRAF1/TRAF2 and BIRC3 mediates anti-apoptotic signaling from TNF receptors by interfering with activation of ICE-like proteases (48). TRAF1/TRAF2 heterodimeric complex is also required for TNF-induced NF-κB activation and protection from TNF-induced death (49, 50). TRAFs are also implicated in IL-17 mediated NFκB activation (51) and CCL20 gene expression (52). As TNF, TWEAK and IL-17 mediated signaling all mediate activation of NF-κB (53), our results suggest an important role for NF-κB activation as a driver of CDDP-induced inflammatory stress response in *BRCA* deficient TNBC cells (**Figure 6I**). Pathway analysis of olaparib-affected DEGs showed downregulation of LXR/RXR activation pathway in both *BRCA1* wild type and mutant TNBC cells, and identified CD14 as a common target (**Supplementary Tables S4**, **S6**). Some effects of PARP inhibition were *BRCA1* mutant TNBC cell-specific such as impact on HIF-1α signaling, cell cycle control by B-cell translocation gene (BTG) and CHK proteins, salvage pathway of pyrimidine synthesis and fatty acid β-oxidation. In *BRCA1* wild type TNBC cells, olaparib affected pathways related to protein citrullation, iNOS signaling, taurine synthesis, and the BER pathway with DNA polymerase γ (Pol γ), an essential player in mitochondrial DNA replication and repair (54–56). Genetic or chemical inhibition of PARP-1 improves mitochondrial DNA content and mitochondrial function, suggesting that PARP-1 can adversely impact mitochondrial DNA maintenance by Pol γ replisome and exacerbates mitochondrial dysfunction (57). Taken together, our data show that while TNBC models with wild type versus mutant *BRCA1* exhibit differences in CDDP-induced cellular response pathways, the CDDP-induced signaling mechanisms remain stable across the isogenic models of OlaR from the same lineage. The *in vitro* data obtained from two isogenic TNBC models support more general conclusions that are of significance to the field of TNBC, however, *in vivo* verification is warranted.

## Conclusions

Our results indicate that TNBC cell responses to CDDP are complex and impact fundamentally different signaling pathways. Notwithstanding this complexity, we have found that PARP inhibition selectively synergizes with cisplatin in TNBC cells with high intrinsic PARP-1 activity, which is independent of their *BRCA1* status. This synergism between PARP inhibition and CDDP could be exploited as a plausible strategy to enhance platinum therapeutic efficacy for both wild type and mutant *BRCA1* TNBCs, and that the PARP-1 activity status could serve as a biomarker for identifying TNBCs that will benefit from PARPi and CDDP combination therapy. Our data also reveal that regardless of their *BRCA1* status, acquisition of OlaR does not alter CDDP sensitivities and the associated signaling mechanisms in the isogenic TNBC counterparts and suggest that platinum-based drugs can have continued therapeutic benefit for TNBC patients after acquiring OlaR. By focusing on CDDP regulated DEG genes in isogenic parental and OlaR TNBC cells, we have identified pathways/genes that could serve as signatures for assessing differential responses of *BRCA1* wild type and mutant TNBC cells to CDDP before and after acquisition of PARPi resistance.

## Data Availability Statement

The datasets generated and analyzed for this study can be found in the gene expression omnibus (GEO) (http://ncbi.nlm.nih.gov/geo/) database through GEO series accession #GSE165914. 

## Author Contributions

MPS and GSW conceived, MS designed, analyzed the results and coordinated the project. AG and AS conducted the experiments and analyzed data, and SK and KG assisted with RNA-seq data analysis. MPS wrote the original draft. GSW, AG, SK, and AS reviewed and edited. All authors contributed to the article and approved the submitted version.

## Funding

This work was supported by a Strategic Research Initiative grant from Karmanos Cancer Institute (to MPS and GSW), NIH R21 CA178117 (to MPS) and 3 Balls Racing (to MPS). The Genome Sciences and Biostatistics Core facilities are supported by NIH Center grant P30 CA022453 to the Karmanos Cancer Institute at Wayne State University.

## Conflict of Interest

The authors declare that the research was conducted in the absence of any commercial or financial relationships that could be construed as a potential conflict of interest.
