# Supplementary material for: Analysis of Adaptive Olaparib Resistance Effects on Cisplatin Sensitivity in Triple Negative Breast Cancer Cells
Source: Front Oncol. 2021 Jul 22;11:694793. doi: 10.3389/fonc.2021.694793 (PMC8339968; doi:10.3389/fonc.2021.694793)

Supplementary Material

# Supplementary Figures and Tables

## Supplementary Figures

#
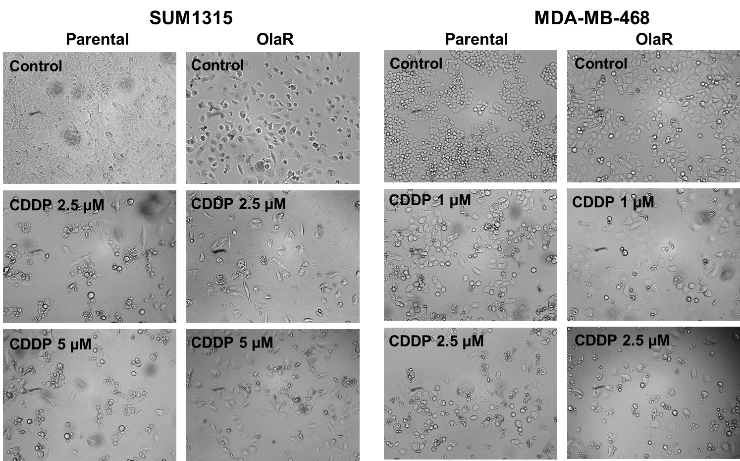
Supplementary Figure 1. Phase contrast micrographs of parental and olaparib resistant (OlaR) SUM1315 and MDA-MB-468 TNBC cells treated with cisplatin (CDDP) compared to their respective controls. Images were taken 24-36 h after treatment. Original magnification ×20.

**Supplementary Figure 2.** Plates showing parental and OlaR SUM1315 and MDA-MB-468 cell colonies stained with crystal violet.

**Supplementary Figure 3.** PARP-1 expressed in OlaR TNBC cells is catalytically inactive. Whole cell lysates were prepared from control or olaparib treated parental SUM1315 cells and their olaparib resistant (OlaR) counterpart and analyzed by western blotting with PARP-1, PAR and loading control GAPDH antibodies.


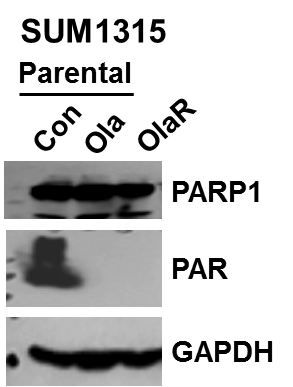


**Supplementary Figure 4.** Heatmap analysis of RNAseq-identified RAD51 and RAD51 family member transcripts expressed in parental and olaparib resistant (OlaR) SUM1315 and MDA-MB-468 cells treated with olaparib, cisplatin (CDDP) or their combination.


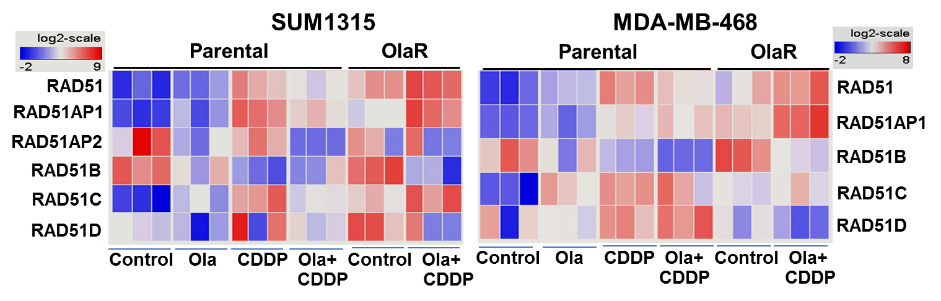


**1.2. Supplementary Tables**

**Supplementary Table 1.** Group specific comparison analysis of SUM1315 colony forming assay data.

**Supplementary Table 2.** Group specific comparison analysis of MDA-MB-468 colony forming assay data.

**Supplementary Table 3.** Gene network related to cisplatin regulated transcripts in parental and olaparib resistant SUM1315 TNBC cells.


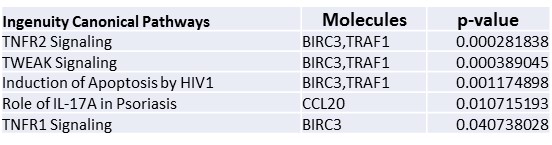


**Supplementary Table 4.** Gene network related to olaparib regulated transcripts in parental and olaparib resistant SUM1315 TNBC cells.


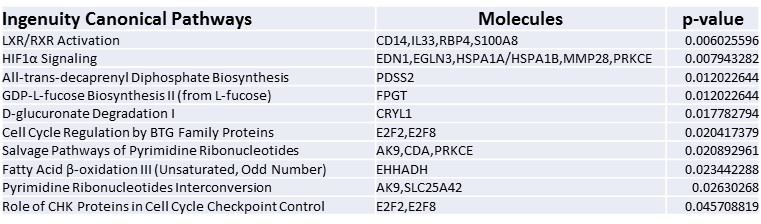


**Supplementary Table 5.** Gene network related to cisplatin regulated transcripts in parental and olaparib resistant MDA-MB-468 TNBC cells.


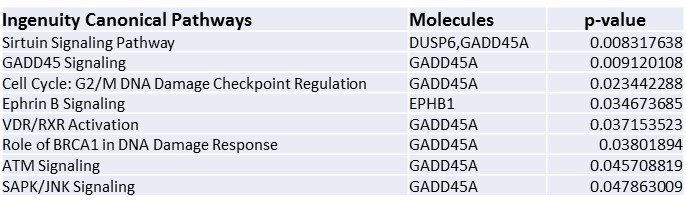


**Supplementary Table 6.** Gene network related to olaparib regulated transcripts in parental and olaparib resistant MDA-MB-468 TNBC cells.


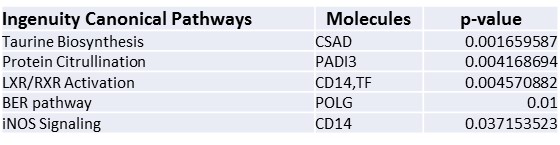

Supplement: Supplementary file 1 [file DataSheet_1.docx]
